# External Iliac Artery Endofibrosis: A Discussion on Two Unique Cases

**DOI:** 10.7759/cureus.44839

**Published:** 2023-09-07

**Authors:** Vincent F Carfagno, Justin Rouintan, Michael A Rucker, David Carfagno

**Affiliations:** 1 Diagnostic Radiology, Midwestern University Arizona College of Osteopathic Medicine, Glendale, USA; 2 Sports Medicine, Scottsdale Sports Medicine Institute, Scottsdale, USA; 3 Biological Sciences, Arizona State University, Tempe, USA

**Keywords:** endurance training athletes' endofibrosis, athletic injuries, ileofemoral bypass, lower-extremity claudication, external iliac artery endofibrosis, inflammatory arteritis, endarterectomy, angioplasty, sports-related injury, iliac artery endofibrosis

## Abstract

Iliac artery endofibrosis (IAE), as the name suggests, involves subintimal fibrosis of the iliac artery. IAE is most commonly associated with competitive athletics, particularly cycling, and remains a rather underappreciated diagnosis in the clinical setting. We present two unique and distinct presentations of IAE in competitive athletes. The first case involves a 38-year-old male cyclist who initially presented with complaints of a bulge at the right groin and acute onset monoplegia and paresthesia associated with exertion of the right lower extremity. This patient was referred to vascular surgery and underwent right common iliac artery and proximal common femoral artery endarterectomy with patch angioplasty and Fogarty embolectomy. Case 2 involves a 50-year-old female triathlete who presented with left lower extremity claudication of a more chronic course, with symptoms beginning approximately four years prior. The pain radiated to her upper thigh and was associated with exertion, restricting her exercise tolerance and return to training. After a diagnosis of IAE was made, she was referred to vascular surgery for a left iliofemoral bypass.

## Introduction

Iliac artery endofibrosis (IAE) is an underrecognized disease resulting from non-atheromatous inflammatory arteritis with resultant subintimal fibrosis of the iliac artery [1]. IAE is more common in competitive athletes, with cyclists in particular being at a greater risk [2,3]. Previous studies have determined a prevalence of IAE between 10% and 20% among competitive cyclists [4].

This report describes two distinct cases of IAE; the first case involves a 38-year-old male cyclist who presented with complaints of intermittent claudication associated with monoplegia and paresthesia at the right lower extremity that occurred with activity. The case remained unique in the patient’s lack of a classical presentation, with an initial complaint of a bulge at the right groin with concern for an inguinal hernia. What also makes this case distinct is the patient’s rather acute onset of symptoms, with symptoms of claudication occurring within two weeks after the patient engaged in a five-day regimen of physical activity, which included strenuous daily cycling. The patient was treated with endarterectomy, patch angioplasty, and Fogarty embolectomy.

The second case involves a 50-year-old female triathlete who presented with left lower extremity pain of a more chronic course. This case, in comparison to case 1, highlights the variance in clinical presentation for IAE, as the patient’s symptoms presented over four years. This patient also reported severe symptoms at onset, raising an initial suspicion for compartment syndrome at the lateral and anterior compartments of the left lower extremity. After treatment with fasciotomy of the respective compartments, her symptoms improved but persisted. With continued concern secondary to the chronic and indolent nature of her symptoms, the patient was referred to vascular surgery and diagnosed with IAE. She was subsequently treated with iliofemoral bypass surgery with improved revascularization at the distal extremity.

## Case presentation

Case 1 concerned a healthy 38-year-old male cyclist with no significant past medical or surgical history who presented with complaints of a new onset bulge at the right groin along with acute onset claudication and associated monoplegia and paresthesia present with movement of the right lower extremity. The patient reported that his entire right lower extremity suddenly started to become weak to the point of immobility within 5-10 minutes of beginning a cycling session. He noted complete resolution of his symptoms with rest. His symptoms began less than two weeks after a five-day course of strenuous physical activity through which the patient was cycling and hiking for long periods of time each day. He reported a long history as a bicyclist and denied having similar symptoms in the past. Physical exam was notable for a protrusion at the right inguinal area during Valsalva without any strength or sensory deficits throughout the bilateral lower extremities. Normal pulses were present throughout. The patient was also noted to have a slight thoracic dextroscoliotic curve and paraspinal hypertonicity throughout the right lumbar region.

**Figure 1:**
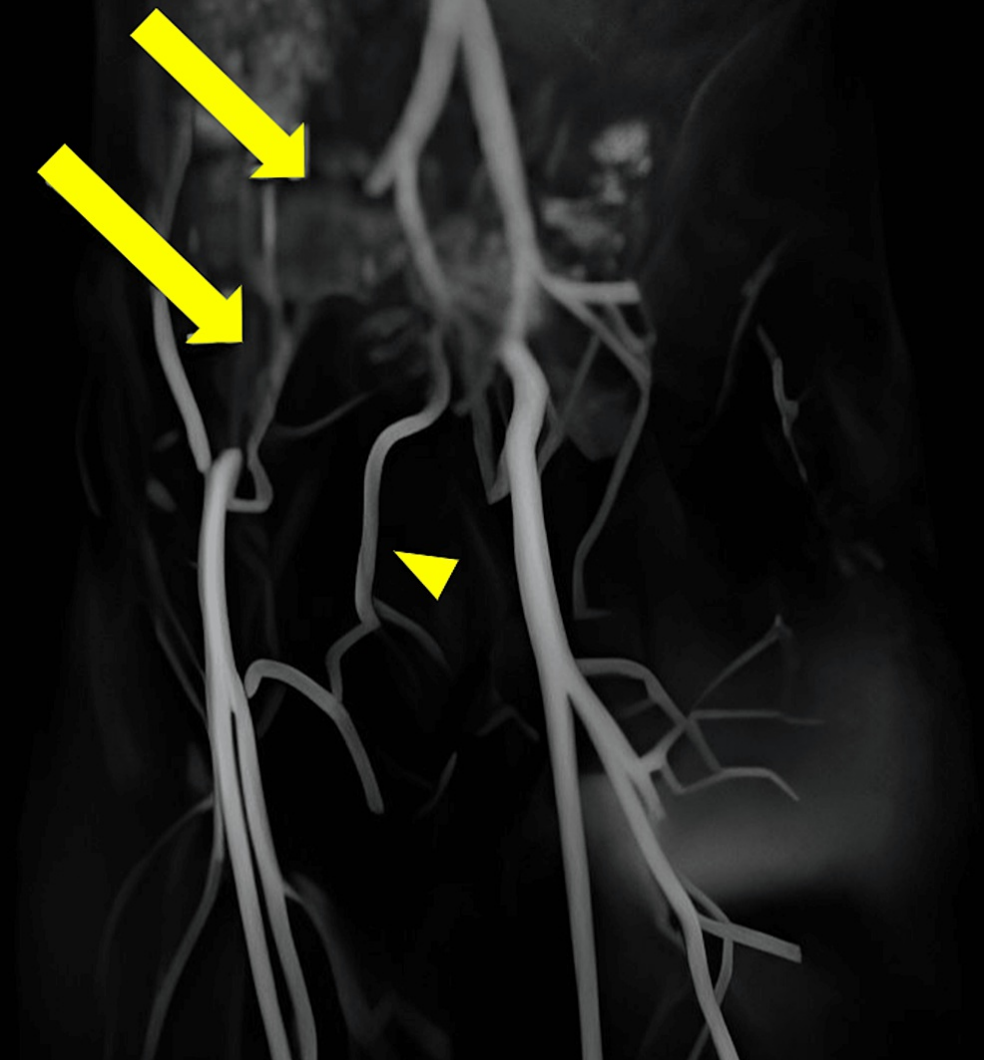
MRA bilateral lower extremities with 3D reconstruction of case 1 MRA of the bilateral lower extremities with 3D reconstruction displaying an 8-cm occlusion extending from the proximal REIA to the common femoral artery (arrows). Collateral flow from the right epigastric and internal iliac arteries can also be visualized (arrowhead).

Due to the acute onset of the patient’s symptoms and concern for a symptomatic inguinal hernia, the initial workup included ultrasound (US) with Doppler of the right inguinal region, which remained without evidence of an inguinal hernia or adenopathy. X-ray (XR) of the lumbar spine was also performed, displaying L5-S1 spondylolysis without significant degenerative changes or fracture. With growing suspicion for IAE, magnetic resonance (MR) of the lumbar spine and pelvis was performed, displaying trace L5-S1 grade I anterolisthesis with trace bilateral L5 pars defects and edema at the dorsal lumbar paraspinal musculature. MR angiography (MRA) exhibited an 8-cm occlusion extending from the proximal right external iliac artery (REIA) to the level of the common femoral artery. MRA with 3D reconstruction was rendered to display the extent of occlusion (arrows, Figure 1). Compensatory collateral flow from the right epigastric and internal iliac arteries was also visualized (arrowhead, Figure 1).

Following these findings, a consult was placed to vascular surgery, and the patient underwent a right common iliac artery and proximal common femoral artery endarterectomy with patch angioplasty and Fogarty embolectomy. No postoperative complications were noted.

Case 2 reports on a 50-year-old female triathlete who presented with chronic left lower extremity pain radiating to the left pelvic region. Her pain was elicited and exacerbated with exercise. Physical examination was unremarkable at rest. The patient reported her symptoms initially began four years prior while participating in a triathlon event. Her pain resolved with abstinence from training; however, she noted persistent and significant symptoms of claudication with physical exertion. Additionally, she manifested signs of compartment syndrome and had experienced a left-sided foot drop shortly after the initial onset of her symptoms, with slight improvement after fasciotomy.

**Figure 2:**
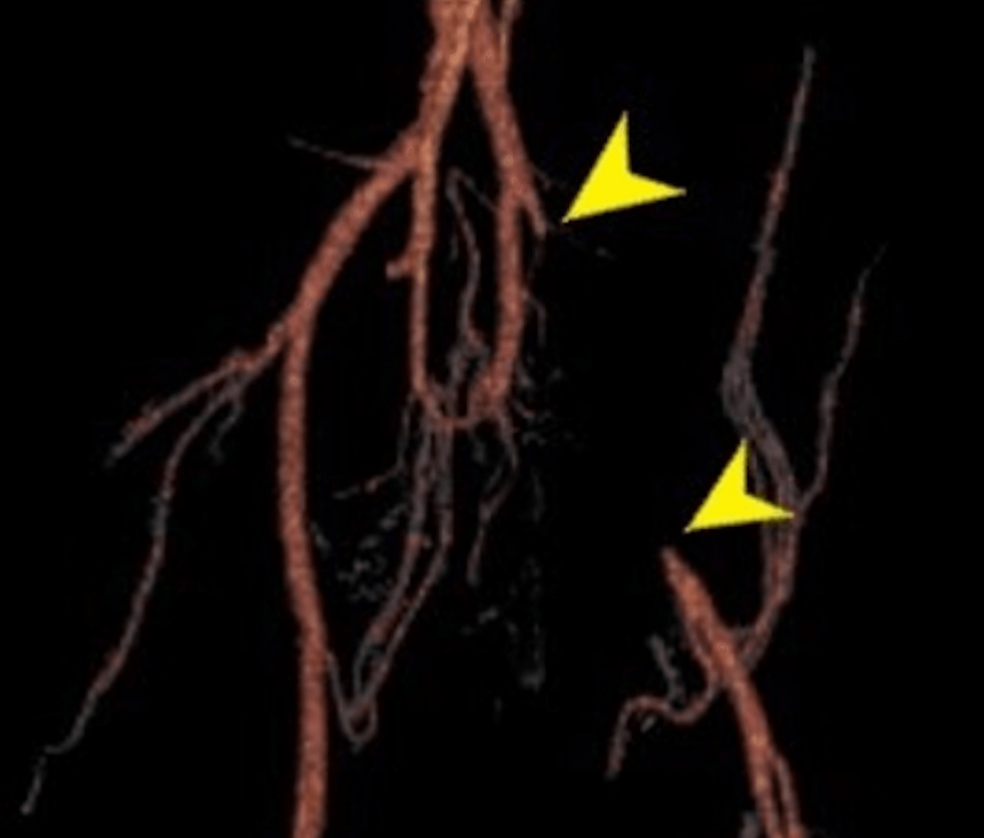
CTA bilateral lower extremities with 3D reconstruction of case 2 CTA of the bilateral lower extremities with 3D reconstruction displaying the extent of atresia stemming from the proximal left external iliac artery.

Two years after symptom onset, the patient received further workup due to persistent claudication with exertion despite treatment with fasciotomy. She was then diagnosed with a small tear in the gastrocnemius and treated with platelet-rich plasma (PRP) therapy. Despite this, her symptoms continued, heightening suspicion of IAE. Further diagnostic imaging, including computed tomography angiography (CTA) of the pelvis, was obtained. CTA with 3D reconstructed imaging led to the discovery of atresia and occlusion of the proximal left external iliac artery (Figure 2).

Following these findings, the patient was referred to vascular surgery for a left iliofemoral bypass with improvement in her symptoms. No postoperative complications were noted.

## Discussion

First described in 1986, IAE is an underrecognized pathology involving intimal layer fibrosis of the iliac artery [1,5]. Resulting from non-atheromatous inflammatory arteritis, IAE is more commonly seen in endurance cyclists, particularly young athletes who are otherwise healthy, though cases have been associated with other athletes, including skiers, runners, and rowers [6-8]. A majority of cases of IAE, around 85%, occur unilaterally with symptoms, including that of lower extremity claudication exacerbated during exercise with significant improvement shortly after a period of rest [6,9].

Cases in the literature describe a rather chronic and indolent onset of symptoms in patients affected by IAE [6,10,11]. Distinctly, case 1 described encompasses a healthy 38-year-old male cyclist presenting with complaints of severe onset claudication less than two weeks after a five-day episode of strenuous exercise. Diagnosis of IAE is complicated by a typically delayed onset of patient symptoms, as well as the rapid resolution of symptoms at rest, the latter of which was seen in patients from both of the cases described, who had normal neurovascular exams without any notable pulse, strength, or sensory deficits. Put simply, patients with IAE may not present with abnormal findings on physical exam due to the rapid resolution of symptoms with rest. It is for this reason that a diagnosis of IAE should remain on the differential in young, otherwise healthy patients who present with a history of claudication. A focused physical exam should be performed, including evaluation of lower extremity strength/sensation and the ankle-brachial index [6]. To account for the resolution of symptoms at rest, a more precise diagnosis of IAE may be achieved with auscultation of the iliac artery in hip flexion and extension [6]. Suspicion of IAE should be followed by diagnostic imaging, including CTA or MRA.

While the underlying pathophysiology remains unclear, IAE is thought to be due to a combination of mechanical derangements such as the repetitive hip hyperflexion associated with cycling along with psoas hypertonicity and the resultant fixation of the iliac artery, all of which may provide a nidus for mechanical trauma of the iliac artery [12]. Treatment of IAE depends on a number of factors, including the intensity of symptoms, the severity of findings on diagnostic imaging, and the overall athletic goals of the patient. Modalities of intervention include conservative management, such as a limitation of aggravating physical activity (i.e., cycling) as well as positional alterations, such as limiting hip flexion, to decrease aggravating mechanics associated with IAE [6]. Again, athletic goals should be considered in patient management; thus, conservative management should be individualized depending on the patient. The benefit of balloon angioplasty in patients with IAE remains under debate, with concerns being a result of the elastic nature of the disease and the tendency for arterial recoil and thus restenosis following intervention with angioplasty techniques [7]. Similarly, stenting of the iliac artery may be of limited benefit, particularly in patients who desire continued endurance training, as stents may increase the risk for friction of the arterial wall, though more research is required to understand the benefits and drawbacks of such interventions in the management of IAE. More recently, analysis of interventions involving the use of endarterectomy seems to provide a high level of patient satisfaction and symptomatic reduction with a rather low risk of complications [13]. Iliofemoral bypass may be reserved for patients presenting with more severe or refractory symptoms.

## Conclusions

IAE is a rather unrecognized condition primarily affecting endurance cyclists. In athletic patients presenting with a history of intermittent lower extremity claudication, whether acute or chronic, suspicion for IAE should remain high as symptomatic onset may vary. As several other cases, including that of this discussion, described, some patients may also present with atypical symptoms, including nonspecific abdominal pain. Physical exam findings may be noncontributory owing to the quick resolution of symptoms with rest. Nonetheless, a prompt neurovascular exam should be performed. Suspicion of IAE should be followed with diagnostic imaging including CTA or MRA of the pelvis and bilateral lower extremities. Treatment of IAE ranges from conservative management to interventional techniques such as endarterectomy with angioplasty. Management should be individualized based on the severity of symptoms and the overall goals of the patient.
